# Abdominal Massage Alleviates Skeletal Muscle Insulin Resistance by Regulating the AMPK/SIRT1/PGC-1α Signaling Pathway

**DOI:** 10.1007/s12013-021-00983-0

**Published:** 2021-05-08

**Authors:** Yiran Han, Zeyuan Lu, Shaotao Chen, Chongwen Zhong, Minghui Yan, Heran Wang, Meng Meng, Mingjun Liu

**Affiliations:** 1 Departments of Acupuncture and Massage, Changchun University of Chinese Medicine, Changchun, 130117 Jilin Province PR China; 2 Department of Pharmacology, School of Pharmaceutical Sciences, Jilin University, Changchun, Jilin Province 130021 PR China

**Keywords:** Abdominal massage, Insulin resistance, Inflammatory cytokines, Adipocytokine, AMPK/SIRT1/PGC-1α

## Abstract

Abdominal massage (AM), a traditional Chinese medicine-based treatment method, has received considerable attention in the recent years. The aim of the present study was to investigate the effect of AM on high-fat diet (HFD)-induced insulin resistance (IR) in comparison with resveratrol (RSV) treatment. Forty-eight male Sprague-Dawley rats were randomly divided into the following four groups: standard chow diet (control group), high-fat diet (model group), HFD + abdominal massage (AM group), and HFD + resveratrol (RSV group). A rat model of IR was established by feeding HFD to rats for 8 weeks followed by treatment with AM or RSV for 4 weeks. The underlying HFD-induced IR molecular mechanisms were studied in rat serum and skeletal muscles. RSV and AM significantly improved glucose intolerance, hyperglycemia, obesity, and significantly reduced lipid accumulation [triglyceride (TC), total cholesterol (TG), low-density lipoprotein cholesterol (LDL-C), and high-density lipoprotein cholesterol (HDL-C)], adipocytokine [free fatty acids (FFA), adiponectin (ADPN)] and serum pro-inflammatory cytokines (IL-6 and TNF-α) secretion. In addition, AM activated the AMPK/SIRT1 signaling pathway in rat skeletal muscle. In conclusion, our results showed that AM could improve IR by regulating the secretion of adipocytokines, pro-inflammatory cytokines as well as related signaling pathways in the skeletal muscle of rats, which might provide insights into development of new treatment methods for the clinical treatment of IR.

## Introduction

Insulin resistance (IR) is a complex metabolic disorder associated with obesity, nonalcoholic fatty liver disease, atherosclerosis, and type 2 diabetes mellitus (T2DM) [1]. Although the precise mechanism of IR remains unclear, the adenosine monophosphate-activated protein kinase/Sirtuin1 (AMPK/SIRT1) pathway is known to play a pivotal role in IR [2–6]. In recent years, increasing evidence has suggested that inflammatory responses, especially those mediated by adipocytokines, play a key role in the development of IR [7, 8]. Adipocytokines are the cytokines released from adipose tissue, including leptin, adiponectin, acylation stimulating protein, and omentin along with tumor necrosis factor (TNF), interleukin-6 (IL-6), and interleukin-1 IL-1, which can block the activation of the AMPK/SIRT1 insulin signaling pathway and induce IR [9, 10].

Weight gain can lead to the development of morbid obesity, which in early stages can be managed through therapy, diet, physical exercise, and cognitive-behavioral therapy, without the need for medication, endoscopy, or surgery [11]. However, considering the difficulty of maintaining long-term calorie-restricted diets, non-pharmacological interventions and lifestyle changes have received considerable attention for the management of weight gain and related disorders [12]. A substantial number of studies have indicated that massage therapy is beneficial in various conditions, such as metabolic disorders, pain syndromes, immune diseases, and aging-related health issues [13]. Moderate massage therapy has been shown to exert positive effects, including pain relief from various syndromes, increased concentration, decreased depression, and enhanced immune function (increased proportion of natural killer cells and natural killer cell activity) [14].

Traditional Chinese medicine massage therapy has been widely used in clinics as an external treatment [15–17]. Different techniques have been used in clinical treatment and in the prevention of several disease conditions with an aim to stimulate body meridian, improve blood circulation, boost metabolism, regulate the endocrine system, improve the function of autonomic nerves, and to eliminate the symptoms of systemic or local discomfort [18–20]. From the perspective of modern medicine, the abdomen is extremely rich in nerve cells and is also termed as “the second brain” [21]. Abdominal massage (AM) is defined as a non-invasive gentle massaging of abdomen performed to treat various conditions, such as obesity, T2DM, fatigue syndrome, and other medical problems [22–25]. AM can promote glucose and lipid metabolism and is shown to be effective in the clinical treatment of insulin-resistant obese patients [26, 27]. In our previous study, we found that AM significantly increased the expression of SIRT1 mRNA in skeletal muscle and triggered strong anti-inflammatory responses in Sprague-Dawley (SD) rats [28]. To the best of our knowledge, this is the first study to evaluate the effect of AM on IR. In the present study, we aimed to investigate the effects of AM on high-fat diet (HFD)-induced IR and the related mechanisms in comparison with resveratrol (RSV) intervention in SD rats.

## Materials and Methods

### Reagents

RSV (80051933) was purchased from Celex Laboratories Inc., and triglyceride (TC), total cholesterol (TG), low-density lipoprotein cholesterol (LDL-C), and high-density lipoprotein cholesterol (HDL-C) were purchased from Nanjing Jiancheng Bioengineering Institute, Nanjing, China (A110-A113); TNF-α (05619b), IL-6 (05621b), adiponectin (ADPN, 03586b), free fatty acids (FFA, 03247b), and GTX ELISA kits were purchased from Yaji Biological Technology Co., Ltd, Shanghai, China; PhosphoPlus ®AMPKα (Thr172) antibody duet (8208) was purchased from Cell Signaling Technology, Inc., Shanghai, China; SIRT1 (13161-1-AP) and PGC-1α (63369-1-LG) primary antibodies were purchased from Proteintech Group Inc, China; GAPDH primary antibody was purchased from Weikesaisi Technology Inc., Wuhan, China; radioimmunoprecipitation assay (RIPA) lysis buffer (WB-0071), horseradish peroxidase (HRP)-conjugated goat-anti rabbit/mouse secondary antibodies (AB-0041, AB-0021) were purchased from Dingguochangsheng Biotechnology Co., Ltd, Beijing, China; RNA extraction kit (R1051) was purchased from Dongsheng Biotech Inc.; and the TransScript^®^ Green Two-Step qRT-PCR SuperMix (AQ201-01) was purchased from Transgen Biotech Inc., Beijing, China.

### Animals and Models

All animal experimental procedures were performed in accordance with the guidelines for care and use of laboratory animals of Changchun University of Traditional Chinese Medicine and approved by the Animal Experimental Ethics Committee (Approval No. 2020194). The rats (8-week-old male SD rats, weighing 180–220 g) were raised by the College of Pharmacy, Jilin University, Changchun, China. Rats had free access to prescribed food and water under standard temperature (24 °C), relative humidity (50%), and light (light–dark cycle) conditions. After subjection to adaptive feeding for 1 week, rats were randomly divided into a normal diet group (NF, *n* = 12) and a high-fat group (HDF, *n* = 36); NF group rats were provided with normal feed (energy density: 3.8 kcal/g; composition: 70% carbohydrate, 20% protein, and 10% fat); HDF group rats were provided with a HFD (energy density: 5.4 kcal/g; composition: 38.5% carbohydrate, 15% protein, and 46.5% fat [29]). Diets were obtained from National Resource Center for Rodent Laboratory Animal. After subjection to 8 weeks of feeding, the glucose infusion rate was determined by performing glucose clamp assay in 36 rats from the HDF group and was found to be significantly lower than the NF group rats. These HDF group rats (*n* = 36) were then randomly divided into the model, RSV, and AM groups (*n* = 12). Twelve rats from the NF group were treated as controls. Animal experiments were conducted in accordance with laboratory animal care and user guidelines from the National Institutes of Health and were approved by the Ethics Committee of Changchun University of Chinese Medicine and Jilin University College of Pharmacy.

### Treatment

Abdominal rubbing, massaging, and acupoint pressing (RN6 Qi Hai, RN4 Guan Yuan, RN12 Zhongwan, and ST25 Tianshu) of rats in AM group were performed for 15 min, once a day, for 4 weeks [28]. Information on the positioning of acupoints was obtained from “The appendix of Experimental Acupuncture.” Before conduction of the treatment, operators were subjected to training using type TPA-II massage measurement instrument (purchased from Yilian Medical Development Co., Ltd, Shanghai) to learn the correct massage technique and application of pressure, (instrument measuring 0.5 kg). The RSV group rats were administered with 260 mg/kg RSV orally once a day, for 4 weeks. Control groups were orally administered with an equal dose of normal saline once a day, for 4 weeks. The weight of the rats and food intake were measured weekly. After the treatment, all rats were anesthetized to obtain blood samples and were sacrificed thereafter. The quadriceps femoris from all rats was removed and stored in liquid nitrogen for further analysis.

### Analyses of Biochemical Indicators

Fasting blood glucose (FBG) levels were measured using the glucose oxidase method (AICARE, China). Serum levels of TC, TG, HDL-C, and LDL-C were measured using a biochemical marker kit. The levels of fasting serum insulin (FINS), FFA, ADPN, TNF-α, and IL-6 were determined by performing ELISA. The homeostasis model assessment of insulin resistance (HOMA-IR) was calculated using the formula HOMA-IR = FBG × FINS/22.5.

### qPCR Analysis

Total RNA from the samples was isolated using an RNA extraction kit according to the manufacturer’s instructions. RNA reverse transcription and amplification were performed using the TransScript^®^ Green Two-Step qRT-PCR SuperMix. mRNA expression was detected using Stratagene Mx3000p (Agilent, USA). SIRT1 (forward: 5′-CAG CAT CTT GCC TGA TTT GTA A-3′, reverse: 5′-TTG GAA TTA GTG CTA CTG GTC TTA C-3′) and PGC-1α (forward: 5′-CATTCAGGAGCTGGATGGCT-3′, reverse 5′-TATGTTCGCGGGCTCATTGT-3′) mRNA expression levels were calculated by using 2^−ΔΔCt^ and compared with those of β-actin (forward: 5′-CGT TGA CAT CCG TAA AGA CCT C-3′, reverse: 5′-TAG GAG CCA GGG CAG TAA TAT-3′) used as an internal control.

### Western Blotting

Briefly, total protein was extracted using the RIPA lysis buffer, and protein concentration was detected by performing the BCA method. Protein (30 mg) was electroblotted onto a polyvinylidene fluoride (PVDF) membrane following separation on 8% SDS-polyacrylamide gel. The PVDF membrane was incubated with blocking solution (5% skim milk) for 1 h and overnight with antibodies (1:500) at 4 °C. Blots were subjected to washing steps using Tris buffer solution (TBS) and incubated with HRP-conjugated secondary antibody (1:5000). Washing steps were subsequently performed using TBS and blots were developed using the electrochemiluminescence method; results were determined using a chemiluminescence imaging system (Bio-Rad, 1708370). The ImageJ software was used for analysis and semi-quantification of the blots.

### Statistical Analysis

Data are presented as mean ± standard deviation (SD). Statistical analyses were performed using SPSS 22.0 and GraphPad Prism 8. Comparison between groups was performed using unpaired two-tailed Student’s *t* test and ANOVA. A *p* value less than 0.05 was considered as statistically significant.

## Results

### AM Alleviates IR in HFD-Fed Rats

**Fig. 1:**
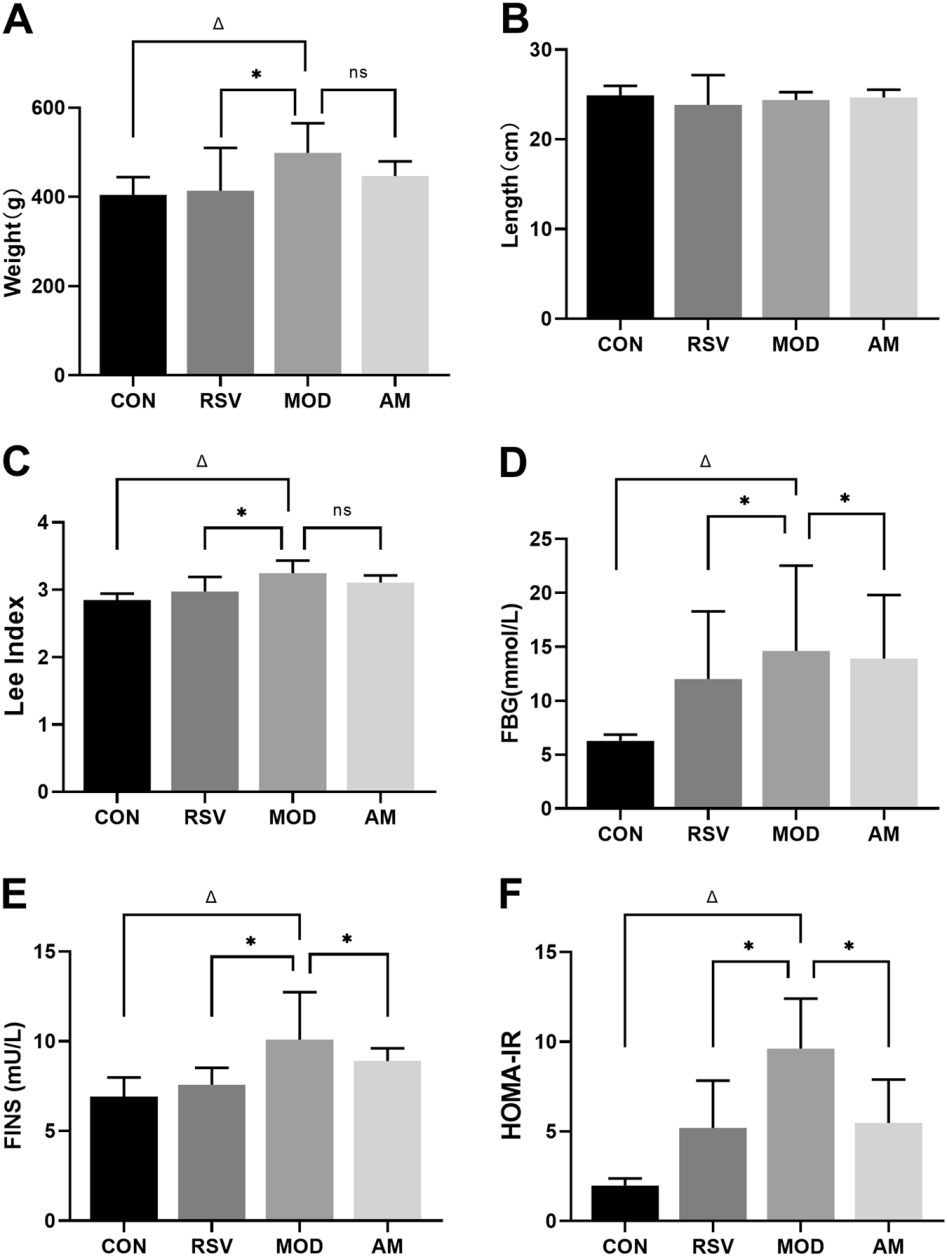
AM alleviates IR in HFD-fed rats. **A** Weight, **B** length, **C** Lee Index, **D** FBG, **E** FINS, and **F** HOMA-IR indices. Data are expressed as means ± SD. Different superscript letters indicate statistically significant differences between the groups (*P* < 0.05). *P < 0.05 represented a significant difference with MOD group; ^Δ^*P* < 0.05 represented a significant difference with CON group. CON control, RSV resveratrol, AM abdominal massage

The growth parameters measured in rats are shown in Table 1 and Fig. 1. Symptom severity was assessed by using the Lee index and HOMA-IR. HFD led to a significant increase in the rat body weight. Among the HFD groups, the Lee index was significantly lower in the RSV and AM groups than the model group (*p* < 0.05). HFD group rats showed significantly greater HOMA-IR after 4 weeks of AM intervention (*p* < 0.05). Although not statistically significant, AM resulted in a weight loss in the AM group rats.

**Table 1:** Am ameliorates the state of HFD-induced IR rats

	CON	RSV	MOD	AM
Weight (g)	404.8 ± 39.21	413.8 ± 96.07^*^	498.70 ± 66.45^Δ^	446.50 ± 33.30
Length (cm)	24.92 ± 1.03	23.83 ± 3.34	24.38 ± 0.87	24.67 ± 0.87
Lee index	2.84 ± 0.10	2.97 ± 0.22^*^	3.24 ± 0.19^Δ^	3.11 ± 0.11
FBG (mmol/L)	6.28 ± 0.58	12.03 ± 6.26^*^	14.63 ± 7.9^Δ^	13.93 ± 5.86^*^
FINS (mU/L)	6.90 ± 1.08	7.59 ± 0.93^Δ^	10.08 ± 2.65^Δ^	8.90 ± 0.72^*^
HOMA-IR	1.98 ± 0.39	5.19 ± 2.64^*^	9.62 ± 2.78^Δ^	5.47 ± 2.42^*^

All resulting data were statistically analyzed using a two-tailed Student’s *t* test. Data are expressed as means ± SD (*n* = 12/groups)

*CON* control, *RSV* resveratrol, *AM* abdominal massage, *FINS* fasting insulin, *HOMA-IR* homeostasis model assessment-insulin resistance

*P < 0.05 was presented a significant difference with MOD group; ^Δ^*P* < 0.05 was presented a significant difference with CON group

### Effect of AM on Serum Lipid Accumulation in HFD-Induced IR Rats

**Fig. 2:**
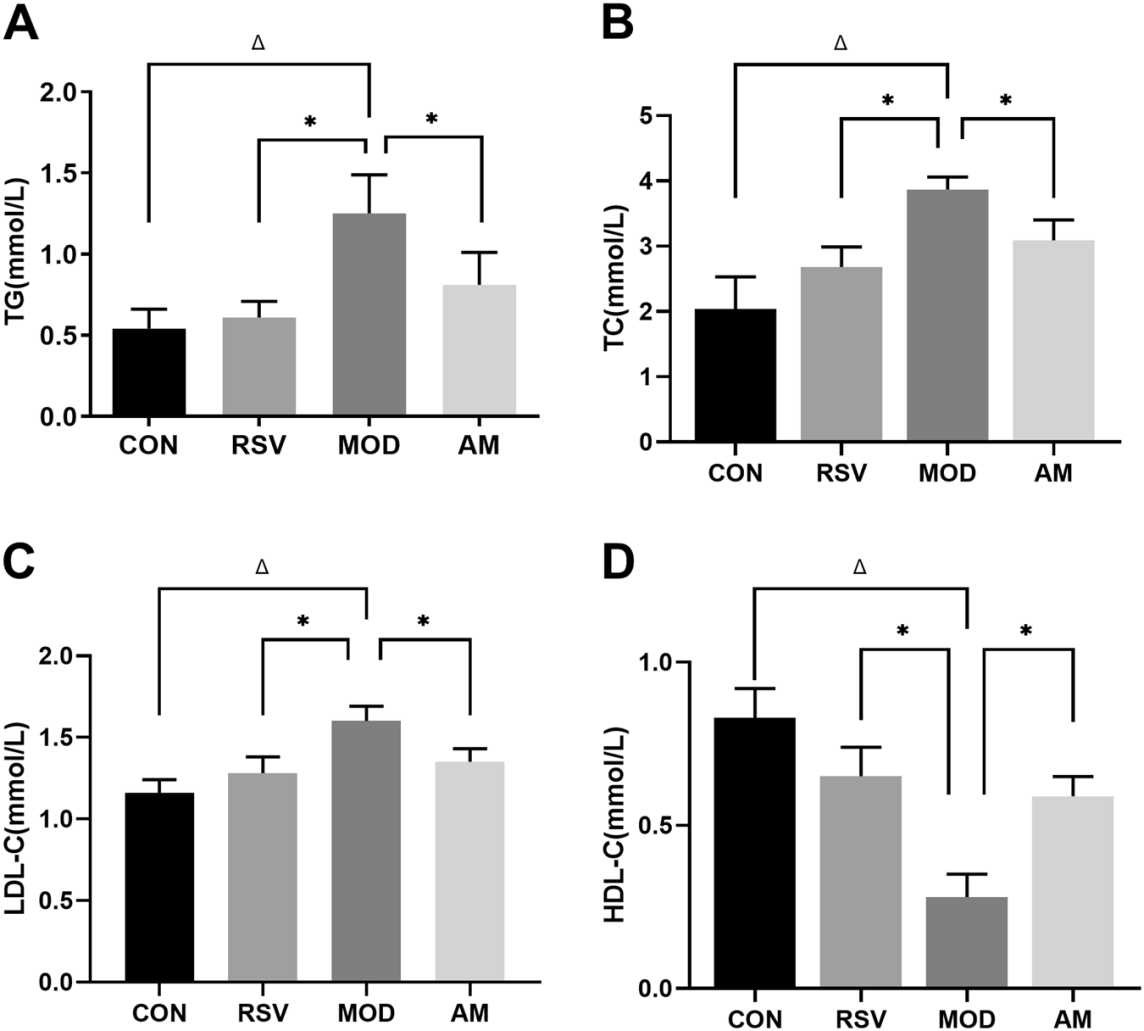
Effect of AM on serum lipid accumulation in HFD-induced IR rats. **A** TG, **B** TC, **C** LDL-C, and **D** HDL-C. Data are expressed as means ± SD. Different superscript letters indicate statistically significant differences between the groups (*P* < 0.05). *P < 0.05 represented a significant difference with MOD group; ^Δ^*P* < 0.05 represented a significant difference with CON group. CON control, RSV resveratrol, AM abdominal massage

As shown in Table 2 and Fig. 2, after 12 weeks HFD feeding, serum TG, TC, and LDL-C levels were found to be significantly higher in the model group as compared to those in the control group rats (*P* < 0.05). In addition, HDL-C levels were found to be lower in the model group rats. Moreover, with the intervention of AM and RSV, the degree of serum lipid accumulation in AM and RSV groups was significantly lower than that in the model group (*P* < 0.05). Our results confirmed that the consumption of a long-term HFD diet leads to the development of dyslipidemia in rats, and continuous AM can effectively alleviate this symptom.

**Table 2:** Effect of AM on serum lipid accumulation of the HFD-induced obese rats

	CON	RSV	MOD	AM
TG (mmol/L)	0.54 ± 0.12	0.61 ± 0.10^*^	1.25 ± 0.24^Δ^	0.81 ± 0.20^*^
TC (mmol/L)	2.04 ± 0.49	2.68 ± 0.31^*^	3.87 ± 0.19^Δ^	3.09 ± 0.31^*^
LDL-C (mmol/L)	1.16 ± 0.08	1.28 ± 0.10^*^	1.60 ± 0.09^Δ^	1.35 ± 0.08^*^
HDL-C (mmol/L)	0.83 ± 0.09	0.65 ± 0.09^*^	0.28 ± 0.07^Δ^	0.59 ± 0.06^*^

The data represent the means ± SD (*n* = 12/groups) of at least three independent experiments. All resulting data were statistically analyzed using a two-tailed Student’s *t* test

*CON* control, *RSV* resveratrol, *AM* abdominal massage, *TC* total cholesterol, *TG* triglycerides, *HDL-C* high-density lipoprotein-cholesterol, *LDL-C* low-density lipoprotein-cholesterol

^*^P < 0.05 was presented a significant difference with MOD group; ^Δ^*P* < 0.05 was presented a significant difference with CON group

### AM Maintains A Balance of Pro-Inflammatory Cytokine Secretion and Adipocytokines in HFD-Induced IR in SD Rats

**Fig. 3:**
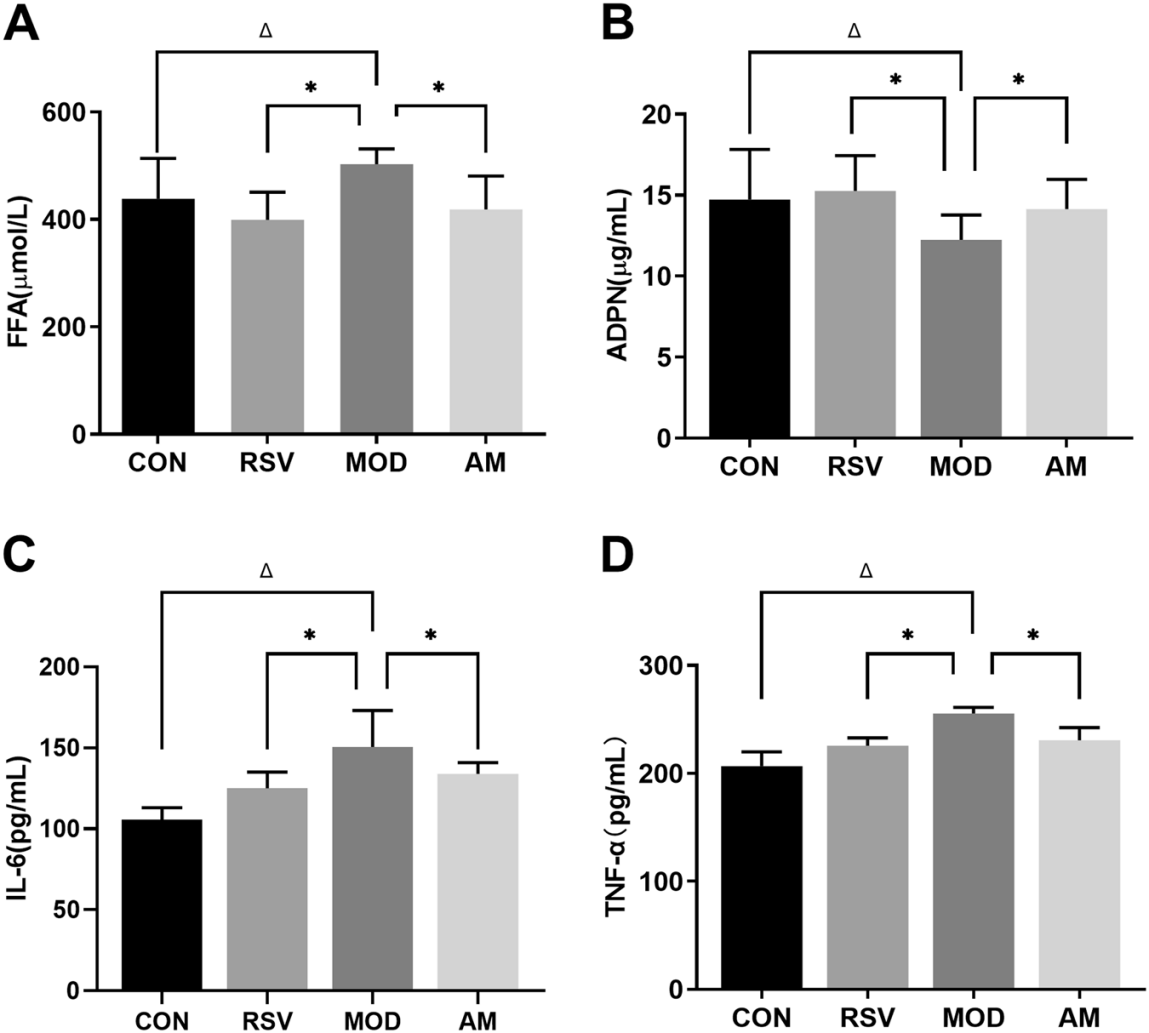
AM maintains a balance of pro-inflammatory cytokine secretion and adipocytokines in HFD-induced IR in SD rats. **A** FFA, **B** ADPN, **C** IL-6, and **D** TNF-α. Data are expressed as means ± SD. Different superscript letters indicate statistically significant differences between the groups (*P* < 0.05). *P < 0.05 represented a significant difference with MOD group; ^Δ^*P* < 0.05 represented a significant difference with CON group. CON control, RSV resveratrol, AM abdominal massage

We analyzed the levels of pro-inflammatory cytokines and adipocytokines, such as TNF-α, IL-6, FFA, and ADPN, in the serum of the rats by performing ELISA. As shown in Table 3 and Fig. 3, the model group had significantly higher serum levels of TNF-α, IL-6, and FFA as compared to the control group (*P* < 0.05), while ADPN was significantly lower (*P* < 0.05) in the model group. After subjection to 4 weeks of AM or RSV treatment, a significant decrease in the serum levels of TNF-α, IL-6, and FFA was observed in AM and RSV group rats as compared to the model group rats (*P* < 0.05). Furthermore, ADPN was found to be significantly higher in the AM and RSV group rats. The difference in the serum levels of FFA and ADPN is also a hallmark of chronic inflammation. Our results showed that HFD-induced IR could stimulate the secretion of pro-inflammatory cytokines and adipocytokines in metabolic disorders. AM reduced the levels of pro-inflammatory cytokines in the serum of obese rats and maintained a balance of pro-inflammatory cytokines and adipocytokines in HFD-induced IR.

**Table 3:** AM maintains a balance of the secretion of pro-inflammatory cytokines and adipocytokines in HFD-induced IR rats

	CON	RSV	MOD	AM
ADPN (μg/mL)	14.73 ± 3.10	15.25 ± 2.19^*^	12.24 ± 1.53^Δ^	14.14 ± 1.84^*^
FFA (μmol/L)	438.30 ± 75.41	399.45 ± 51.22^*^	502.86 ± 28.26^Δ^	418.89 ± 61.64^*^
TNF-α (pg/mL)	206.57 ± 13.23	225.67 ± 7.29^*^	255.27 ± 5.91^Δ^	230.52 ± 11.94^*^
IL-6 (pg/mL)	105.70 ± 7.37	125.13 ± 9.97^*^	150.51 ± 22.64^Δ^	133.98 ± 6.89^*^

The data represent the means ± SD (*n* = 12/groups) of at least three independent experiments. All resulting data were statistically analyzed using a two-tailed Student’s *t* test.

*CON* control, *RSV* resveratrol, *AM* abdominal massage, *FFA* free fatty acids, *ADPN* adiponectin, *TNF-α* tumor necrosis factor-α, *IL-6* Interleukin- 6

*P < 0.05 was presented a significant difference with MOD group; ^Δ^*P* < 0.05 was presented a significant difference with CON group

### AM activates the AMPK/SIRT1/PGC-1α signaling pathway in skeletal muscle of HFD-induced IR rats

**Fig. 4:**
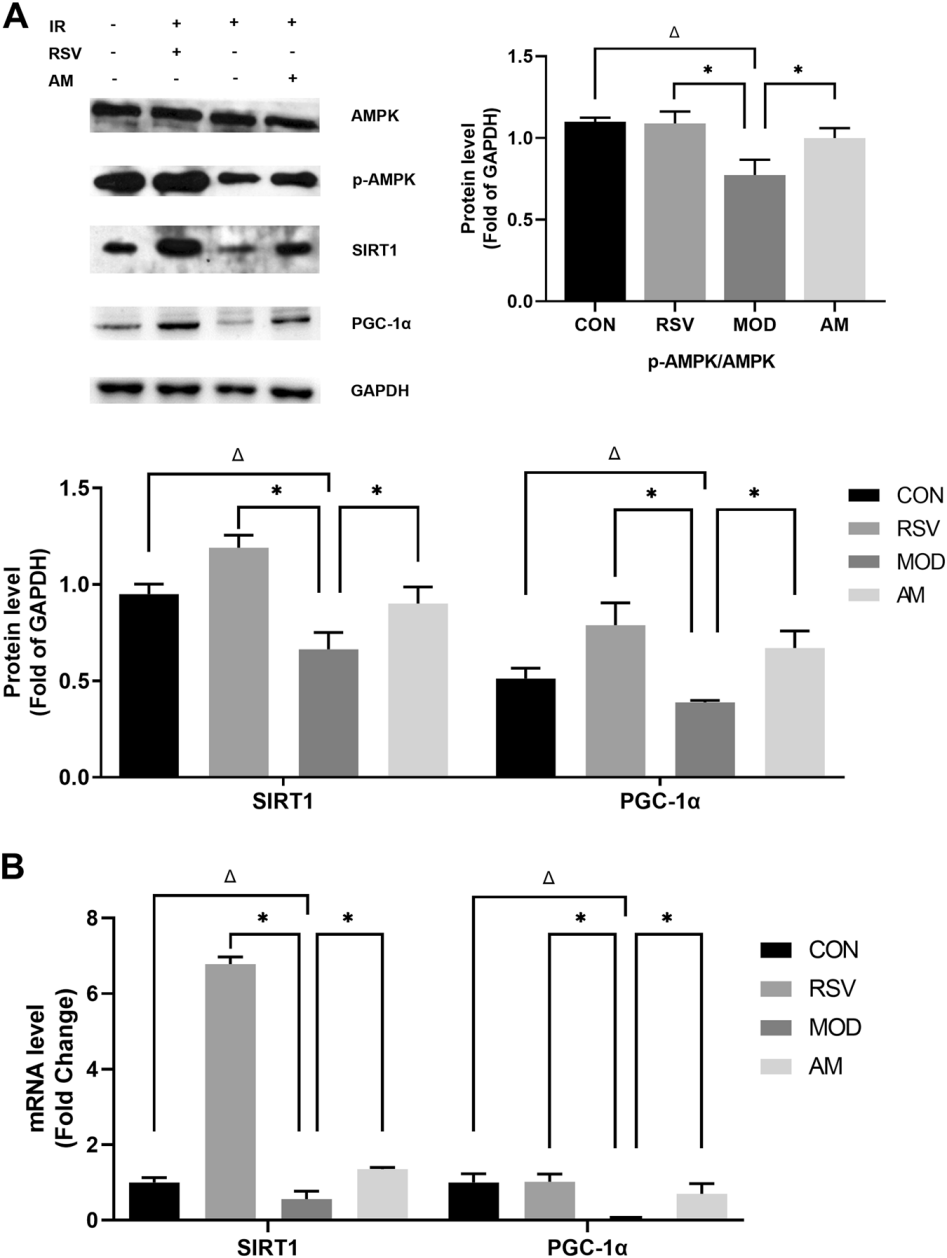
AM activates the AMPK/SIRT1/PGC-1α signaling pathway in skeletal muscle of HFD-induced IR rats. **A** The protein expression of AMPK, p-AMPK, SIRT1, and PGC-1α in skeleton muscle of IR rats. **B** The mRNA expression of SIRT1 and PGC-1α in skeleton muscle of IR rats. Data are expressed as means ± SD. Different superscript letters indicate statistically significant differences between the groups (*P* < 0.05). *P < 0.05 represented a significant difference with MOD group; ^Δ^*P* < 0.05 represented a significant difference with CON group. CON control, RSV resveratrol, AM abdominal massage

The AMPK/SIRT1/PGC-1α pathway is an important signaling pathway that mediates the pathophysiological process of IR in obesity. In our study, we examined the effect of AM on AMPK regulation in skeletal muscles obtained from HFD-induced obese rats. As depicted in Fig. 4A, HFD feeding in rats markedly decreased p-AMPK, SIRT1, and PGC-1α protein and mRNA levels in the skeletal muscle of model group rats (*P* < 0.05). However, persistent AM or RSV treatment reversed this phenomenon by upregulating AMPK phosphorylation levels and by subsequently restoring SIRT1 and PGC-1α protein and mRNA levels.

## Discussion

In the present study, we investigated the effects of AM on HFD-induced IR.

To the best of our knowledge, this is the first study to evaluate the effect of AM on IR. We aimed to investigate the role of AM in mitigating disordered lipid metabolism in the skeletal muscle of obese rats. Our results showed that both AM and RSV alleviated HFD-induced IR. These results were consistent with those reported by previous studies [30, 31]. It has been reported that free fatty acids with endocrine and local effects (paracrine and autocrine) can affect insulin sensitivity. For example, several factors, such as TNF-α, IL-6, leptin, ADPN, IL-1β, resistin, retinol-binding protein, and visfatin, are closely related to IR occurrence [32]. In this study, we examined the levels of adipocytokines and pro-inflammatory cytokines in rat serum by performing ELISA. We aimed to study the effect of AM on lipid metabolism and inflammation in the serum of HFD-induced IR in rats. Our results showed that the serum levels of TC, TG, LDL-C, HDL-C, FFA, ADPN, IL-6, and TNF-α were abnormal in model group rats. All the above-mentioned changes indicated that HFD-induced IR rats developed lipid metabolism disorder and inflammation. However, the related adipocytokines and pro-inflammatory cytokines were shown to be positively regulated in the rats treated by AM. Our results indicate that AM can improve the lipid metabolism disorder and IR-induced inflammation in obese rats.

In addition, chronic inflammation caused by obesity is an important cause of IR [33]. In the case of overnutrition, body stores excess energy in the form of fat with the formation of TG, or with an ectopic deposition in the muscle, liver, placenta, and other tissues, thus leading to obesity [34]. Skeletal muscle is one of the most important peripheral insulin target sites [35]. As reported in several studies, TG deposition in skeletal muscle is one of the pivotal markers of IR in body [36, 37]. Thus, when IR occurs, abnormal glucose and lipid metabolism in skeletal muscle are common. AMPK reduces lipid ectopic accumulation by decreasing circulating glucose and fatty acid levels, thus increasing insulin sensitivity [38, 39]. AMPK not only regulates fat generation, but also reduces FFA concentration in the blood, which has an ameliorating effect on lipid abnormalities in obesity [40, 41]. There is an increasing evidence that SIRT1 regulates glucose and lipid metabolism through its deacetylase activity [42]. As reported in several studies, activated SIRT1 improves insulin sensitivity in the liver, skeletal muscle, and adipose tissue, and confers protection to pancreatic beta-cell function and quality[43, 44]. SIRT1 not only plays an important role in cell proliferation, differentiation, senescence, and apoptosis, but also demonstrates a role in regulating glucose and lipid metabolism, inflammatory response, oxidative stress, tumor formation, and cellular metabolic regulation under different conditions of nutritional stress [45]. AMPK and SIRT1 regulate PGC-1α activity through phosphorylation and deacetylation. Many biological processes, such as mitochondrial biology and fatty acid oxidation, are regulated by the AMPK/SIRT1/PGC-1α signaling pathway. It also promotes fat oxidation and provides energy for the maintenance of lasting muscle contraction and energy supply and is thus closely associated with the development of IR [46]. Our results showed that AM increased the expression of p-AMPK, SIRT1, and PGC-1α in the skeletal muscle of rats, and this indicated that AM activated the AMPK/SIRT1/PGC-1α signaling pathway.

As a representative of external therapy commonly used in traditional Chinese medicine, massage therapy can treat patients’ endocrine disorders, enhance patients’ resting metabolic rate, increase energy consumption, and promote the oxidative utilization of stored fat in the body [47, 48]. It can also enhance blood circulation of the adipose tissue, especially subcutaneous fat, and reduce accumulation. It can also reduce appetite and promote gastrointestinal motility [17, 49]. Currently, there are no specific clinical drugs or treatment methods available for treating obesity and other metabolic disorders. Therefore, there is an urgent need to develop a safe, effective, eco-friendly, and simple clinical therapy for the treatment of obesity. In recent years, the progress witnessed in the treatment of IR in patients with obesity indicates that comprehensive traditional Chinese medicine is gaining prominence as a new therapeutic strategy with increased research focus [50]. Our previous studies [28, 51, 52] have found that AM can significantly improve weight, body mass index, and other indicators of obesity in animal models and obese patients. In this study, our results showed that continuous AM could decrease the secretion of lipids and pro-inflammatory cytokines in the serum of IR rats and could reduce inflammation.

## Conclusion

Taken together, our study proved that AM can help regulate lipid metabolism and reduce inflammation. Our findings suggested that the regulation of IR by AM might be related to the regulation of AMPK/SIRT1/PGC-1α-mediated lipid metabolism and pro-inflammatory factor secretion. These findings provide novel insights into the development of AM as a potential therapy for the improvement of IR-related metabolic disorders, such as T2DM. However, the exact mechanism by which AM results in the treatment of IR-associated complications in other peripheral tissues remains to be elucidated. To this end, further refined studies are warranted to determine its potential for clinical application in obesity treatment.
